# Renoprotection Induced by Aerobic Training Is Dependent on Nitric Oxide Bioavailability in Obese Zucker Rats

**DOI:** 10.1155/2021/3683796

**Published:** 2021-09-28

**Authors:** Rodrigo Vanerson Passos Neves, Hugo de Luca Corrêa, Ivo Vieira de Sousa Neto, Michel Kendy Souza, Fernando Costa, Anderson Sola Haro, Lysleine Alves Deus, Andrea Lucena Reis, Herbert Gustavo Simões, Rosângela Vieira Andrade, Cláudio Oliveira Assumpção, Whitley Stone, Jonato Prestes, Elaine Cristina Vieira, Rita de Cássia Marquetti Durigan, Vinicius Cruzat, Thiago S. Rosa

**Affiliations:** ^1^Graduate Program in Physical Education, Catholic University of Brasília, Brasília, Brazil; ^2^Laboratory of Molecular Analysis, Graduate Program of Sciences and Technology of Health, Universidade de Brasília, Distrito Federal, Brazil; ^3^Department of Nephrology, Federal University of São Paulo, São Paulo, Brazil; ^4^Graduate Program in Genomic Sciences and Biotechnology, Universidade Católica de Brasília, Brasília, Brazil; ^5^Department of Physical Education, Federal University of Ceará, Ceará, Brazil; ^6^School of Kinesiology, Recreation, and Sport, Western Kentucky University, Bowling Green, KY, USA; ^7^Faculty of Health, Torrens University Australia, Brisbane, Australia

## Abstract

Aerobic training (AT) promotes several health benefits that may attenuate the progression of obesity associated diabetes. Since AT is an important nitric oxide (NO^−^) inducer mediating kidney-healthy phenotype, the present study is aimed at investigating the effects of AT on metabolic parameters, morphological, redox balance, inflammatory profile, and vasoactive peptides in the kidney of obese-diabetic Zucker rats receiving L-NAME (N(omega)-nitro-L-arginine methyl ester). Forty male Zucker rats (6 wk old) were assigned into four groups (*n* = 10, each): sedentary lean rats (CTL-Lean), sedentary obese rats (CTL-Obese), AT trained obese rats without blocking nitric oxide synthase (NOS) (Obese+AT), and obese-trained with NOS block (Obese+AT+L-NAME). AT groups ran 60 min in the maximal lactate steady state (MLSS), five days/wk/8 wk. Obese+AT rats improved glycemic homeostasis, SBP, aerobic capacity, renal mitochondria integrity, redox balance, inflammatory profile (e.g., TNF-*α*, CRP, IL-10, IL-4, and IL-17a), and molecules related to renal NO^−^ metabolism (klotho/FGF23 axis, vasoactive peptides, renal histology, and reduced proteinuria). However, none of these positive outcomes were observed in CTL-Obese and Obese+AT+L-NAME (*p* < 0.0001) groups. Although Obese+AT+L-NAME lowered BP (compared with CTL-Obese; *p* < 0.0001), renal damage was observed after AT intervention. Furthermore, AT training under conditions of low NO^−^ concentration increased signaling pathways associated with ACE-2/ANG1-7/MASr. We conclude that AT represents an important nonpharmacological intervention to improve kidney function in obese Zucker rats. However, these renal and metabolic benefits promoted by AT are dependent on NO^−^ bioavailability and its underlying regulatory mechanisms.

## 1. Introduction

The gaseous signaling molecule nitric oxide (NO^−^) plays a key role in the body, including renal hemodynamics regulation. NO^−^ bioavailability is regulated by an increase of NO^−^ production via NO synthase (NOS) activity, using L-arginine as a substrate, and/or lower NO^−^ degradation [1, 2]. In obesity-related disorders, such as type 2 diabetes (T2DM), the renal NO^−^ bioavailability is impaired, which increases renin secretion, intraglomerular pressure, tubuloglomerular feedback, and renal sodium reabsorption, while renal blood flow perfusion is reduced [3–5]. These effects induce kidney damage leading to the development of diabetic nephropathy, chronic kidney disease (CKD), and cardiovascular disease associated with the pathogenesis of systemic hypertension [2, 6, 7].

Physical activity and/or exercise training is the cornerstone nonpharmacological adjunct for treating and preventing several medical conditions, including obesity-related disorders [8, 9]. It is already established that exercise improves glucose homeostasis by reducing chronic inflammation and oxidative stress [3, 9, 10]. For instance, Ito et al. showed that regular running exercise upregulated NOS expression, while suppressing NADPH oxidase and *α*-oxoaldehydes in the kidneys, which at least in part improved renal protection in the early progression of diabetic nephropathy in Zucker diabetic fatty rats [11]. Similar results were also observed by other authors, where aerobic exercise during 4 weeks promoted protective effects in a diabetic kidney by reducing renal oxidative stress and inflammation in female Wistar rats [12]. Exercise also decreases mitochondrial oxidative stress by reducing the mitochondrial permeability transition pore (MPTP) [13, 14]. Interestingly, this effect regulates the action of vasoactive peptides, such as angiotensin 1-7, bradykinin, and vasopressin, which are associated with improvements in NO^−^ bioavailability. Restoring NO^−^ bioavailability has been considered a key mechanism to improve endothelial dysfunction in obesity-related disorders [15–19].

In endothelial dysfunction, the elevated vascular oxidative stress decreases NOS expression, especially the endothelial NOS isoform (eNOS), thus reducing NO^−^ bioavailability [20, 21]. Notably, exercise training decreased renal oxidative stress and systemic blood pressure even when chronic inhibition of eNOS was experimentally induced by N(omega)-nitro-L-arginine methyl ester (L-NAME) [22, 23]. L-NAME has been widely applied for several decades in basic and clinical research as an antagonist of NOS [24–29], reducing NO^−^ bioavailability in tissues such as the kidney [30, 31]. However, some studies have shown that while exercise improves systemic blood pressure under reduced eNOS activity, it may increase renal injury in animal models [23, 32]. Thus, exercise has clear health benefits; however, the renal effects of aerobic exercise training (AT) and biological action of NO^−^ in obesity-related conditions, such as T2DM, require further elucidations.

Renoprotection is a complex phenomenon involving the cross-talk between multiple mechanisms and NO^−^ signaling pathways. Functional assessments, morphological, biochemical, and molecular approaches might aid understanding of the complete picture of cellular adaptation in response to AT in a kidney structure. A mechanistic framework of these responses could provide valuable insights for therapeutic approach development and treatment guidance to attenuate kidney diseases and disorders, besides mapping of key biomarkers in physiological responses inherent to obesity conditions. Since exercise is an important NO-inducer mediating kidney function, the present study is aimed at investigating the effects of 8 weeks of AT on metabolic parameters, morphological parameter, redox balance, inflammatory profile, and vasoactive peptides in the kidney of obese-diabetic Zucker rats receiving L-NAME. We hypothesized that Zucker rats exposed to L-NAME display no improvements in glucose homeostasis, blood pressure, kidney structure, redox balance, and inflammatory state.

## 2. Materials and Methods

### 2.1. Animals

Forty 6-wk-old male homozygous obese (*fa^−^*/*fa^−^*) and lean (*Fa^+^/fa^−^*) Zucker rats obtained from the Animal House were used in this research and maintained under a 12 h light/dark cycle (lights on at 07 : 00) at room temperature of 22 ± 2°C and relative humidity of 55 ± 10%. After one week of acclimation, the animals were distributed into four groups (*n* = 10, each): sedentary lean rats (CTL-Lean), sedentary obese rats (CTL-Obese), AT-trained obese rats without blocking the NOS (Obese+AT), and AT-trained obese rats with NOS blocked (Obese+AT+L-NAME). During the study, animals were maintained in separate cages (4-3 animals per cage according to their phenotype), fed a standard chow (NUVILAB CR1, Nuvital® Nutrients, Curitiba, Brazil), and received water *ad libitum*. All procedures followed the NIH Guide for the Care and Use of Laboratory Animals (U.S. National Research Council, Washington D.C., USA) [33, 34] and international principles for research involving animals (ARRIVE 2.0) [35]. The present study was approved by the Animal Research Ethics Committee (protocol number: 007/19).

### 2.2. Determination of the Exercising Lactate

Lactate threshold (LT) was assessed 48 h after the first insulin tolerance test (ITT) [36]. All animals running at 6 m/min speed for 10 min three times per week for two weeks to familiarize the rats with the AT training ergometer (treadmill developed for rats customized model, AVS Projects, Brazil).

The electric shock (5 mV) was conducted according to the NIH Guide for Care and Use of Laboratory Animals [33, 34]. Stimulus was used in familiarization, physical examinations, and exercise training. This familiarization protocol was intended to reduce the animals' stress when performing the required exercise tasks. Intensity started at 6 m·min^−1^ and was increased by 2 m·min^−1^ for each 3 min stage until exhaustion. In addition to lactate evaluation, the investigators also determined maximum running velocity (Vmax) achieved during the last stage in the graded exercise test. Vmax was obtained in the incremental test, as the velocity of the last complete stage was supported by the animal [37].

Blood (5 *μ*L) obtained from the tail was collected at the beginning of the test (rest) and the end of each complete stage using a portable lactometer (Accutrend® PLUS-Roche, USA) to quantify lactate concentration. After local antisepsis with 70% alcohol, 25 *μ*L of blood was collected from a small incision in the distal tail portion using a calibrated capillary tube. The blood sample was rapidly deposited in Eppendorf® microtubes (0.6 mL), containing 50 *μ*L of 1% sodium fluoride (NaF), and stored at −80°C for further biochemical analysis [38].

To determine LT by the visual inspection method, lactate levels were plotted on individual graphs (lactate versus exercise intensity). The evaluators determined the lactate curve's tipping point as the moment when lactate exponentially increased relative to the exercise intensity. These incremental tests were previously described by Rosa et al. applied in all rats pre- and posttraining [38].

These incremental tests were also used to evaluate the intensity closest to the maximal lactate steady state (MLSS) and reduce the number of sessions required to determine the MLSS. The criterion used to identify MLSS was a blood lactate range of up to 0.5 mmol·min^−1^ during the last 10 min of exercise proposed for rats [38].

### 2.3. Aerobic Training

The study began with treadmill running familiarization, according to a protocol adapted from Copp et al. [39]. Rats ran at 6 m/min, 10 min per day, 5 days a week, during 2 weeks on a treadmill with individual lanes and electrical stimulation at the rear (customized model, AVS Projects, Brazil). 48 h after the last MLSS assessment, rats in the AT groups started the exercise protocol in the same treadmill of the MLSS: 60 min at the measured 100% MLSS and 0% grade. Training occurred five days per week for eight weeks as described by Rosa et al. [38].

### 2.4. Inhibition of Nitric Oxide Synthases by L-NAME

Animals assigned to the Obese+AT+L-NAME group were orally supplemented with L-NAME administered in the drinking water at a 5 mg/100 mL concentration for 8 weeks following the protocol published by Bayls et al. [40] L-NAME is a nonselective inhibitor of NOS, but at this concentration, it is a selective inhibitor of eNOS. L-NAME was administered at the beginning of AT and maintained until the end of the protocol.

### 2.5. Glucose and Insulin Tolerance Tests

Glucose and insulin tolerance tests were conducted in all animals two days after [36] the beginning of the 8 weeks of L-NAME supplementation and exercise. The glucose tolerance test (GTT) was performed using an intraperitoneal glucose solution (2 g/kg body weight) at baseline and at posttraining (48 h after isolated cage time). Glycaemia was determined at 0, 15, 30, 60, and 120 min after the glucose injection. ITT was also performed at baseline and posttraining (48 h after GTT). The ITT required an injection of 0.1 U/kg of recombinant regular insulin Humulin® intraperitoneally. Glycaemia was measured at 0, 5, 10, 15, 20, and 30 min. Glycaemia in both GTT and ITT tests was measured using an AccuCheck Performa Roche®. Fasting plasma insulin (FPI) was measured a rat-sensitive enzyme-linked immunosorbent assay (ELISA) kit (Millipore Corporation, Billerica, MA). Homeostasis model assessment (HOMA) for insulin resistance (HOMA-IR) and HOMA for *β*-cell function (HOMA-*β*) were calculated, as previously described by Ito et al. [11]: HOMA‐IR : [fasting insulin (ng/mL) × fasting glucose (mg/dL)]/405; HOMA‐*β* : [fasting insulin (ng/mL) × 20]/[fasting glucose (mg/dL)–3.5].

### 2.6. Blood Pressure Measurement

Systolic blood pressure (SBP) was measured for all animals before and after the AT intervention. Blood pressure measurement was performed according to Neves et al. [41]. Briefly, the SBP was measured using the tail-cuff method with the rats under the conscious condition with the PowerLab system (ADInstruments, Inc., Sydney, Australia). This tail-cuff method is a sensitive and accurate approach for the noninvasive measurement of BP in conscious SHR [41]. SBP was measured once a week at the same time each day (between 6:00 and 8:00 p.m.) to allow the animals to become adapted to the procedure.

### 2.7. Euthanasia and Tissue Harvest

One at a time, after 48 h of the MLSS, the animals were euthanized as described by Neves et al. [42], and the kidneys were harvested. The right kidney was weighed and separated for histology, and the left kidney was utilized for molecular and biochemical analyses. Samples obtained from the left kidney were also frozen at -80°C and used for mRNA and protein determination. The kidney was weighted and corrected by the tibial length [43].

### 2.8. Renal Gene Expression of eNOS, iNOS, and Inflammatory Profile

The total RNA was extracted according to the TRIzol method described by Chomczynski and Sacchi [44]. A NanoDrop® spectrophotometer (ND-1000; NanoDrop Technologies Inc., Wilmington, DE, USA) was used to quantify the RNA concentrations and determining the absorbance rate of 260–280 nm. To assess the eNOS and iNOS gene expression in the kidney, a total of 100-350 ng of RNA extracted from each sample was converted to cDNA (final volume 20 *μ*L) using GoTaq® Probe RT-qPCR (Promega-Cat. A6120X) based on the manufacturer's protocol. The standard cycling conditions to perform reverse transcription and amplification of samples were as follows: (a) reverse transcription (1 cycle)—45°C for 15 minutes; (b) reverse transcriptase inactivation and GoTaq® DNA Polymerase activation (1 cycle)—95°C for 2 minutes; (c) denaturation (40 cycles)—95°C for 15 seconds; and (d) annealing and extension (40 cycles)—60°C for 1 minute. The following TaqMan probes were used for the determination of A-actin (Rn00667869m1), eNOS (Rn02132634s1), and iNOS (Rn00561646m1).

Inflammatory profile was measured in kidney samples following homogenization. Total proteins were extracted from tissues using phosphate-buffered saline (PBS 1%, pH 7.4) supplemented with a protease inhibitor cocktail (Roche, Germany). Tumor necrosis factor-alpha (TNF-*α*), c-reactive protein (CRP), interleukin 10 (IL-10), and interleukin 4 (IL-4) were assessed by commercial ELISA kits manufactured by R&D Systems (USA). Interleukin 17a (IL-17a) was measured by a rat-sensitive IL-17a ELISA kit manufactured by Invitrogen (Thermo Fischer Scientific, MA, USA). For this kit, sensitivity was established at 1.0 pg/mL, and the reproducibility intra-assay and interassay were 8.5% and 7.6% CVs, respectively. Interleukin 18 (IL-18) was measured using a rat IL-18 ELISA kit manufactured by Abcam (São Paulo, Brazil), the sensitivity was <1 pg/mL, and the reproducibility intra- and interassays were lower than 6.2% and 7.2% CVs. The kidney protein content was analyzed by the Bradford method [45]. Standard curves for each cytokine were generated using serial dilutions of the mediators supplied, with each sample titrated by linear interpolation. All samples were determined in duplicate to guarantee reliability.

### 2.9. Histological Analyses

The right kidney was weighted and sectioned in the frontal plane (5 *μ*m); then, it was fixed with 10% formaldehyde (10 mM phosphate buffer, pH 7.4) and embedded into paraffin. The blades were stained with hematoxylin-eosin (HE) for the evaluation of structural changes. Sections were visualized at a magnification of 200x (Leica DM1000, Wetzlar, Germany, 20 × objective and 10 × oculars). Approximately 50 glomeruli and renal tubule sections were evaluated. Each photomicrograph was delimited near the outer edges; individual glomeruli were surrounded to determine the glomerular area using AxioVision Rel, 4.8 software (Carl Zeiss, IL, USA). The tubular diameter was measured at the widest cross-sectional point of the captured images using the same software [46].

### 2.10. Biochemical Analyses

Renal tissue was defrosted, sectioned, and transferred to ice-cold 0.9% NaCl containers and homogenized in 0.1 mol/L Tris-HCl buffer (pH 7.4). The levels of thiobarbituric acid-reactive substances (TBARS), glutathione (GSH), glutathione disulfide (GSSG), superoxide dismutase (SOD), catalase (CAT), NO^−^, and trolox equivalent (TE; total antioxidant capacity) were analyzed following the manufacturer's specifications. Protein carbonyls, 3-nitrotyrosine (3-NT), klotho, angiotensin 1 converting enzyme (ACE-1), angiotensin 2 converting enzyme (ACE-2), angiotensin II type-1 receptor (AT_1_R), bradykinin (BK), and FGF23 were determined using rat protein carbonyl, rat 3-NT, rat klotho, rat ACE-1, rat ACE-2, rat AT_1_R, rat BK, and rat FGF23 ELISA kits, respectively. The overall intra- and interassays CVs for markers were in a range of 2 to 15% (MyBioSource, Inc., San Diego, USA). Asymmetric dimethylarginine (ADMA) and angiotensin II (ANGII) concentrations were determined in duplicate using the rat ADMA (CVs: intra- and interassays were <15%) and rat ANGII (CVs: intra- and interassays were <8% and <10%, respectively) ELISA kits (Cusabio Technology LLC, MD). Angiotensin II type-2 receptor (AT_2_R), angiotensin 1-7 [ANG-(1-7)], and vasopressin dosages were performed using the rat AT_2_R (CVs: intra- and interassays were <10% and <12%, respectively) ELISA kit (LifeSpan BioSciences, Inc., Seattle, USA), rat ANG-(1-7) ELISA kit, and rat vasopressin ELISA kit; the overall intra- and interassays CVs for ANG-(1-7) and vasopressin were in a range of 5 to 16% (Biocompare, Inc., South San Francisco, USA). Two days after the last AT session, each animal was relocated to an individual cage, food and water were offered *ad libitum*, and 24 h urine samples were collected. Urinary creatinine levels were evaluated in duplicate (CVs: intra- and interassays < than 5%) by the colorimetric method using a kit (LABTEST Diagnostics, São Paulo, Brazil). Urinary 8-isoprostane was analyzed in duplicate by ELISA; the overall intra- and interassays CVs were 11.7 and 16.4, respectively (Cayman Chemical, Ann Arbor, MI). Urinary protein excretion (UPE) was assessed in duplicate by colorimetric assay using a Sensiprot Labtest kit with CVs: intra- and interassays < 10% (Centerlab Ltda, São Paulo, Brazil). Urinary excretion of 8-hydroxydeoxyguanosine (8-OHdG) was measured in duplicate (CVs: intra- and interassays < 12%) using an ELISA kit (Northwest Life Science Specialties, LLC; Vancouver, WA).

### 2.11. Renal Mitochondrial Swelling and ROS Production

**Figure 1:**
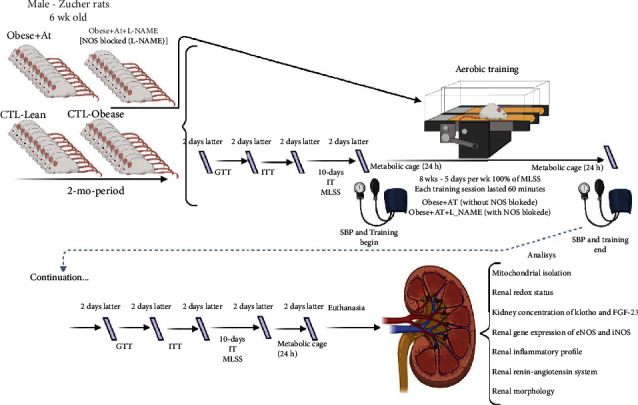
Experimental design. CTL-Lean: sedentary lean rats; CTL-Obese: sedentary obese rats; Obese+AT: obese rats who underwent aerobic training without blocking of nitric oxide synthases; Obese+AT+L-NAME: obese rats who underwent aerobic training with blocking of nitric oxide synthases; L-NAME: N(*ω*)-nitro-L-arginine methyl ester; GTT: glucose tolerance test; ITT: insulin tolerance test; IT: incremental test; MLSS: maximal lactate steady state; BP: blood pressure; AT: aerobic training; eNOS: endothelial nitric oxide synthases; iNOS: inducible nitric oxide synthases. Male Zucker rats were selected at six weeks old. Rats belonging to the Obese+AT+L-NAME group received L-NAME 8 weeks prior to the beginning of the glycemic homeostasis tests and throughout the experimental design. All rats were analyzed GTT, ITT, IT, MLSS, and BP pre- and posttraining. All these analyses were performed with an interval of two days between them. After two days of the initial physical tests, the animals of trained groups were submitted to AT groups ran for 60 min at an intensity equivalent to 100% of MLSS, with 0% grade, 5 days/wk/8 wk. Afterward, 48 h of the last training session, each rat was isolated in a cage for 24 h urine collection. Euthanasia was performed after two days of the last physical fitness tests. The tibia length was measured (renal weight was corrected for tibial length). The kidney was collected for the biomolecular analyses in the mitochondria, redox and inflammatory profiles in the renal homogenate, and weighed. In addition, evaluations were performed on molecules related to the metabolism of renal nitric oxide, as well as biomarkers of renal health klotho/FGF axis23 and vasoactive peptides.

To isolate mitochondria from the renal tissue, we used the differential centrifugation protocol described by Pedersen et al. [47]. Subsequently, internal mitochondrial membrane permeability studies were performed by the mitochondrial osmotic swelling estimated by the decrease of the absorbance at 540 nm with the aid of a Hitachi U-2000 spectrophotometer (Hitachi, Tokyo, Japan), in the presence of CaCl_2_ at a concentration of 50 *μ*M. These assays were adapted from Kowaltowski et al. [48]. The amount of mitochondrial protein was determined by the Biuret method [49]. Changes of 2′,7′-dichlorodihydrofluorescin diacetate fluorescence were used to assess the production of ROS by mitochondria. 2′,7′-Dichlorofluorescein (DCF) fluorescence was accompanied at 503/529 nm excitation/emission wavelength pair in a Fluorescence Spectrophotometer Hitachi F-2500 (Hitachi, Tokyo, Japan). As previously described by Mello et al., ROS production was represented by relative fluorescence units (RFU) [50]. The experimental design of this study is presented in Figure 1.

### 2.12. Statistical Analysis

For *F* tests, the sample power was calculated from an alpha of 5% (*p* < 0.05) and the power of 95% for large effect size. Results of the a priori power analysis (*p* < 0.05 and 95% power) were conducted using GPower® indicating the need of at least 8 rats per group. All data were presented as the mean ± SD. Normality and homoscedasticity were evaluated using the Shapiro-Wilk and Levene tests, respectively. A 2 × 2 (group × time interaction) ANOVA followed by Tukey's Honestly Significant Difference (Tukey HSD) post hoc test was used to identify group × time differences (*p* < .05) for the following variables BW, SBP FBG, FPI, GTT, ITT, HOMA-IR, HOMA-*β*, MLSS, and Vmax. One-way ANOVA with Tukey HSD post hoc test were used to identify differences (*p* < 0.05) between the groups for mitochondrial swelling and ROS production by mitochondria, renal and urinary parameters, inflammatory and redox profiles, klotho, FGF23, renin-angiotensin system, BK, vasopressin, and renal morphology. Analyses were performed using GraphPad Prism 6.0 software (GraphPad Software, Inc., CA, USA).

## 3. Results

At the baseline, body weight (BW) was higher in the obese groups when compared to the lean group. All groups gained weight from baseline to posttest. As expected, the SBP was higher in the obese groups before and after training than in the CTL-Lean group. Baseline SBP was lower in the CTL-Obese and Obese+AT groups when compared to the Obese+AT+L-NAME group. Post hoc analyses indicated that the Obese+AT and Obese+AT+L-NAME groups had lower SBP than the CTL-Obese group posttraining. The CTL-Obese group was the only group in this study that increased SBP when compared to baseline. Pretraining FBG was higher in the Obese+AT+L-NAME group than in the CTL-Obese and Obese+AT groups, which reinforces the impact of the decrease in the action of NO^−^ on the increase in blood glucose. Both CTL-Obese and Obese+AT had higher levels of FBG than CTL-Lean. Following the analysis of group versus time interaction, it was observed that AT decreased FBG in obese rats when compared to CTL-Obese. However, the sum of lower levels of NO^−^ plus AT aggravated the increase in FBG. Furthermore, AT decreased hyperinsulinemia (FPI), glucose intolerance (GTT), and insulin resistance (ITT and HOMA-IR) and improved the pancreatic beta function marker (HOMA-*β*) compared to CTL-Obese. The reduced levels of NO^−^ levels promoted by L-NAME are associated with AT appears to increase glucose intolerance (GTT), insulin resistance (ITT and HOMA-IR), and reduction in pancreatic beta function (HOMA-*β*) caused by obesity and T2DM. AT increases aerobic endurance (MLSS) and maximal running speed (Vmax) in obese Zucker rats. However, the obesity and T2DM observed in Zucker rat cause a decrease in MLSS and Vmax verified in the pretraining period and after the end of the experimental protocol. Data are shown in Table 1.

**Figure 2:**
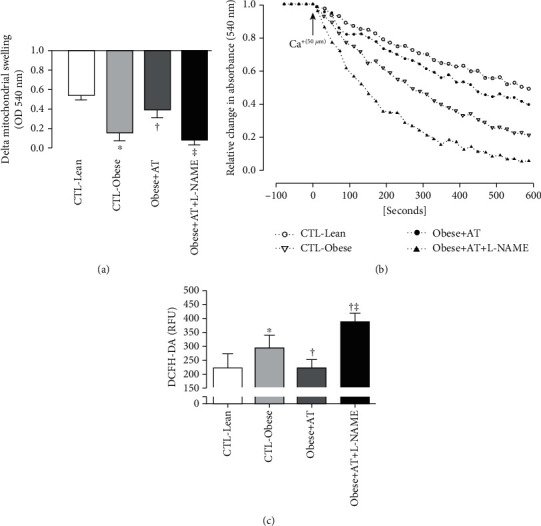
Swelling and production of species reactive to oxygen by the renal mitochondria. Data are presented as the mean ± SD. CTL-Lean: sedentary lean rats; CTL-Obese: sedentary obese rats; Obese+AT: obese rats who underwent aerobic training without blocking of nitric oxide synthases; Obese+AT+L-NAME: obese rats who underwent aerobic training with blocking of nitric oxide synthases. (a) Delta mitochondrial swelling. (b) The figure illustrates the fall in the absorbance of the renal mitochondria (swelling) against the insult with calcium chloride of the groups. (c) Production of species reactive to oxygen by the renal mitochondria. The one-way ANOVA followed by Tukey's post hoc test was adopted. The one-way ANOVA followed by Tukey's post hoc test was adopted. ^∗^*p* < 0.0001*vs.* CTL-Lean; ^†^*p* < 0.0001*vs.* CTL-Obese; ^‡^*p* < 0.0001*vs.* AT.

Kidney mitochondrial swelling at the concentration of CaCl_2_ 50 *μ*M was higher in the obese groups when compared to the CTL-Lean group. However, the Obese+AT group attenuated the mitochondrial swelling when compared to the CTL-Obese and Obese+AT+L-NAME groups (Figures 2(a) and 2(b); *p* < 0.0001). Production of ROS by renal mitochondria (i.e., evaluated by an indicator of oxidative stress DCFH-DA) was lower in the CTL-Lean and Obese+AT groups when compared to the CTL-Obese and Obese+AT+L-NAME groups (Figure 2(c); *p* < 0.0001).

**Figure 3:**
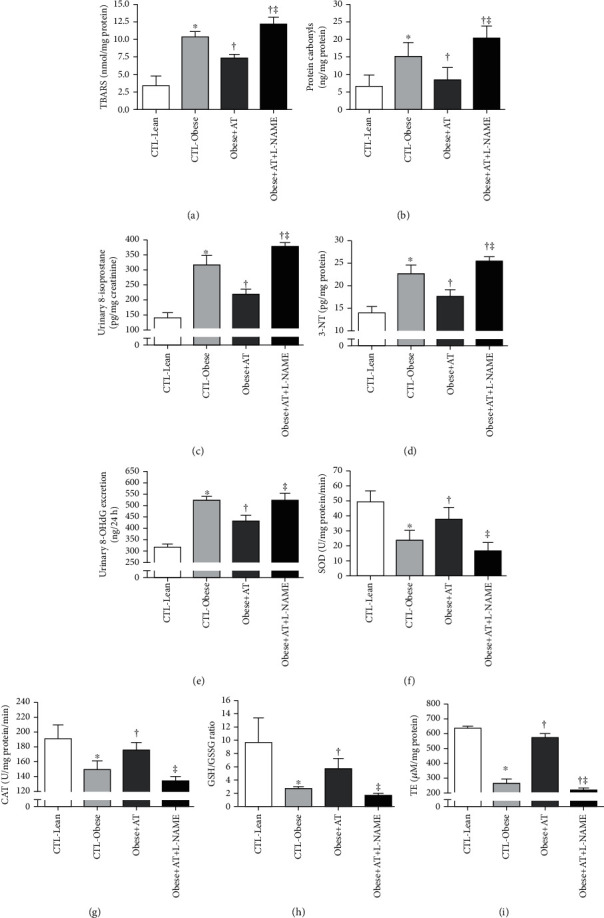
Renal redox balance. Data are presented as the mean ± SD. CTL-Lean: sedentary lean rats; CTL-Obese: sedentary obese rats; Obese+AT: obese rats who underwent aerobic training without blocking of nitric oxide synthases; Obese+AT+L-NAME: obese rats who underwent aerobic training with blocking of nitric oxide synthases; TBARS: thiobarbituric acid-reactive substances; 3-NT: 3-nitrotyrosine; 8-OHdG: 8-hydroxydeoxyguanosine; SOD: superoxide dismutase; CAT: catalase; GSH/GSSG ratio: glutathione/glutathione disulfide ratio; TE: trolox equivalent. The one-way ANOVA followed by Tukey's post hoc test was adopted. ^∗^*p* < 0.0001*vs.* CTL-Lean; ^†^*p* < 0.0001*vs.* CTL-Obese; ^‡^*p* < 0.0001*vs.* Obese+AT.

The obesity of the Zucker rat causes oxidative stress in different cellular structures, as observed by the increase in lipid peroxidation markers (TBARS and urinary 8-isoprostane), protein (3-NT) and mitochondrial DNA (urinary 8-OHdG) oxidation, and dysregulation in antioxidant enzymes more present in the cytoplasm (CAT), in the mitochondria (SOD), and in the general antioxidant balance (GSH/GSSG ratio and TE-Trolox equivalent) (CTL-Obese, Obese+AT, and Obese+AT+L-NAME compared to CTL-Lean; *p* < 0.0001). AT reduces these oxidative damages in these different subcellular structures (Obese+AT compared to CTL-Obese and Obese+AT+L-NAME; *p* < 0.0001), but in the absence of NO^−^, AT exacerbates these damages (Obese+AT+L-NAME compared to CTL-Obese; *p* < 0.0001). Values are shown in Figure 3.

**Figure 4:**
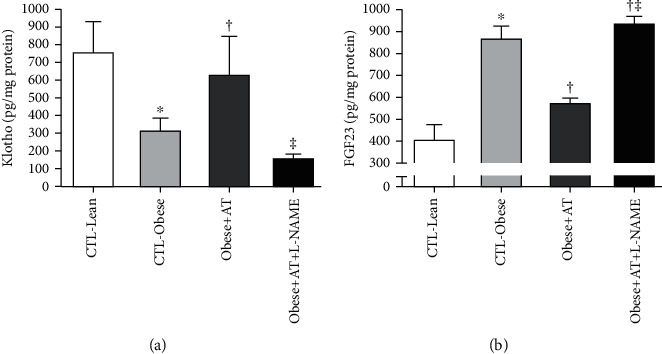
Renal klotho/FGF23 axis. Data are presented as the mean ± SD. CTL-Lean: sedentary lean rats; CTL-Obese: sedentary obese rats; Obese+AT: obese rats who underwent aerobic training without blocking of nitric oxide synthases; Obese+AT+L-NAME: obese rats who underwent aerobic training with blocking of nitric oxide synthases; FGF23: fibroblast growth factor 23. The one-way ANOVA followed by Tukey's post hoc test was adopted. ^∗^*p* < 0.05*vs.* CTL-Lean; ^†^*p* < 0.0001*vs.* CTL-Obese; ^‡^*p* < 0.0001*vs.* Obese+AT.

AT increases Klotho and decreases FGF23 in Zucker rats (Obese+AT compared to CTL-Obese and Obese+AT+L-NAME; *p* < 0.0001), thus combating the imbalance in the Klotho/FGF23 axis caused by obesity (CTL-Obese, Obese+AT, and Obese+AT+L-NAME compared to CTL-Lean; *p* < 0.0001), but under a low concentration of NO^−^, AT amplifies this lack of control (Obese+AT+L-NAME compared to CTL-Obese; *p* < 0.0001). Data are shown in Figure 4.

**Figure 5:**
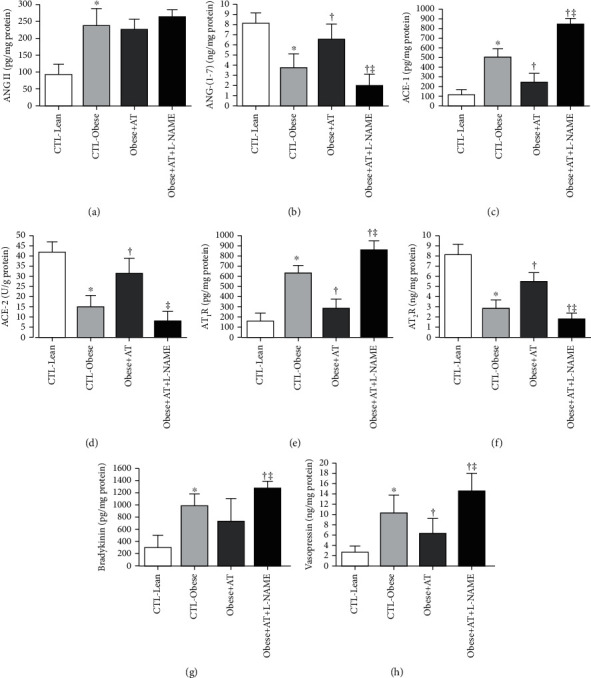
Kidney vasoactive peptides. Data are presented as the mean ± SD. CTL-Lean: sedentary lean rats; CTL-Obese: sedentary obese rats; Obese+AT: obese rats who underwent aerobic training without blocking of nitric oxide synthases; Obese+AT+L-NAME: obese rats who underwent aerobic training with blocking of nitric oxide synthases; ANGII: angiotensin II; ANG-(1-7): angiotensin 1-7; ACE-1: angiotensin-converting enzyme 1; ACE-2: angiotensin-converting enzyme 2; AT_1_R: angiotensin II type-1 receptor; AT_2_R: angiotensin II type-2 receptor. The one-way ANOVA followed by Tukey's post hoc test was adopted. ^∗^*p* < 0.0001*vs.* CTL-Lean; ^†^*p* < 0.0001*vs.* CTL-Obese; ^‡^*p* < 0.0001*vs.* Obese+AT.

In the obese Zucker rat, ACE-1/ANGII/AT_1_R pathway hyperactivation and ACE-2/ANG-(1-7)/AT_2_R reduction were observed, which are important to induce glomerular and tubulointerstitial lesions (CTL-Obese, Obese+AT, and Obese+AT+L-NAME compared to CTL-Lean; *p* < 0.0001). However, AT decreases ACE-1, AT1R, and vasopressin and increased ACE-2, ANG-(1-7), and AT2R in obese Zucker rats, which culminated in a decrease in kidney damage (Obese+AT compared to CTL-Obese and Obese+AT+L-NAME; *p* < 0.0001). Additionally, AT concomitant with a decrease in NO^−^ worsens these pathways of the renin-angiotensin system in the kidney (Obese+AT+L-NAME compared to CTL-Obese; *p* < 0.0001). All these results are presented in Figure 5.

In obese Zucker rats, an increase in proteinuria was observed (CTL-Obese, Obese+AT, and Obese+AT+L-NAME when compared to CTL-Lean), which was reduced by the action of AT (Obese+AT compared to CTL-Obese and Obese+AT+L-NAME). However, the addition of AT with NO^−^ deficiency to proteinuria has been extended (Obese+AT+L-NAME compared to CTL-Obese). Zucker rats with obesity displayed a renal decrease of NO^−^, gene expression of eNOS, and anti-inflammatory cytokines, such as IL-10 and IL-4. In turn, they presented an increase in the concentration of ADMA, gene expression of iNOS, and proinflammatory cytokines, including TNF-*α*, CRP, IL-17a, and IL-18 (CTL-Obese, Obese+AT, and Obese+AT+L-NAME compared to CTL-Lean). Nonetheless, AT reverses these molecular adverse scenarios (Obese+AT compared to CTL-Obese and Obese+AT+L-NAME). Again, these mechanistic dysfunctions are aggravated when AT and NO^−^ deficiency are combined (Obese+AT+L-NAME compared to CTL-Obese). Data are shown in Table 2.

**Figure 6:**
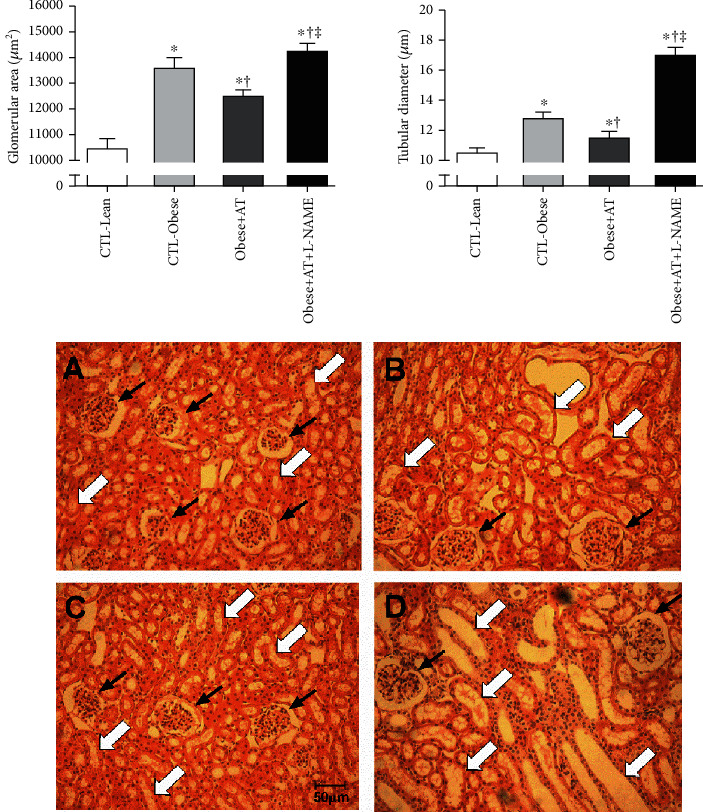
Representative photomicrographs of the kidney. Hematoxylin-eosin staining. (a) CTL-Lean; (b) CTL-Obese; (c) Obese+AT; (d) Obese+AT+L-NAME. Black arrows indicate glomerulus, and white arrows indicate renal tubules. Magnification, 200x. ^∗^*p* < 0.0001*vs.* CTL-Lean; ^†^*p* < 0.0001*vs.* CTL-Obese; ^‡^*p* < 0.0001*vs.* Obese+AT.

Figure 6 shows that obese Zucker rats have an increase in a glomerular area and tubular diameter (CTL-Obese, Obese+AT, and Obese+AT+L-NAME compared to CTL-Lean; *p* < 0.0001). AT minimizes such structural damage to glomeruli and renal tubules (Obese+AT compared to CTL-Obese and Obese+AT+L-NAME). However, given the low concentration of NO^−^, a glomerular disorganization is observed, with an expressive increase in its area and tubulointerstitial disarrangement (Obese+AT+L-NAME compared to CTL-Obese). Representative photomicrograph of renal histology is shown in Figure 6.

## 4. Discussion

The present study was conducted to investigate AT's effects in obese Zucker rats with low levels of NO^−^ induced by L-NAME. Our results support the initial hypothesis, showing that chronic effects of AT upon the kidney are dependent on renal NO^−^ bioavailability. Although exercise is a well-known NO^−^ inducer associated with several health-related benefits, we have demonstrated that AT increased kidney injury in obese Zucker rats receiving L-NAME. Furthermore, we observed that these animals exhibited no improvements in well-known health parameters associated with chronic response to exercise, such as glucose homeostasis, blood pressure decreases, inflammatory, and redox balance control. On the other hand, obese rats not treated with L-NAME have shown a reduction in kidney injury, mitochondrial dysfunction, blood pressure, fasting glucose, and proteinuria, as well as improves oxidative stress, inflammatory profile, Klotho/FGF23 axis, and renin-angiotensin system.

Obesity and associated chronic diseases, such as T2DM, are well-known leading causes of renal injury and CKD. For instance, chronic hyperglycemia is considered a central mechanism in the pathogenesis promoting metabolic changes such as redox imbalance, which increases the intrarenal inflammation [51] and mitochondrial osmotic changes leading to CKD [52, 53]. Moreover, hyperglycemia induce an increase in the flow of electrons in the transport chain, increasing the production of mitochondrial ROS [51]. Thus, elevated ROS production activates cell death downstream pathways, causing kidney damage and inflammation [53]. Dada and Sznajder reported that high levels of proinflammatory cytokines are associated with mitochondrial swelling, which alters permeability transition pore [54]. On the other hand, AT modulates mitochondrial membrane permeability linked to a reduction of oxidative stress and inflammation, hence attenuating the effects of renal damage associated to obesity. Interestingly, Rosa et al. observed an increase in aerobic capacity and maximum speed in obese Zucker rats after eight weeks of AT [38]. In the present study, the lack of NO^−^ bioavailability in the Obese+AT+L-NAME group reduced aerobic capacity improvements. This is possibly due to the fact that NO^−^ plays an important role in controlling several physiological mechanisms that influence the delivery of oxygen to the cells, such as regulating muscle blood flow, mitochondrial biogenesis and respiration, relaxation/contraction cycle, and glucose uptake [55].

Aerobic exercise training has been broadly used as a nonpharmacological antihypertensive therapy decreasing the risk of metabolic and cardiovascular disease [56]. In most research papers, these positive outcomes are associated with improvements in endothelial dysfunction and a decreased eNOS activity [57]. In the present study, trained obese rats not treated with L-NAME have shown less vulnerable nephrons to damage and more capable of coping with oxidative stress due to increased antioxidant enzymes involved in the protection process while reducing prooxidant agents (Figure 3). Several animal and human studies have shown that AT promotes protection via redox balance [11, 17, 19, 58, 59]. Indeed, NO^−^ is an essential contributor to reduce artery stiffness, vascular tone control, and sympathetic activity in response to flow-mediated shear stress [60]. Thus, NO^−^ is a critical determinant of the magnitude of the exercise-induced blood pressure response [19]. Additionally, NO^−^ is also known to have beneficial effects on active myocardial relaxation through diastolic volume maintenance at high heart rates during exercise training [57].

In the present study, AT modulated the Klotho-FGF23 axis, which is considered a key mechanism for renal integrity maintenance [61, 62]. Physiologically, proteins filtered in glomerular capillaries are captured in the proximal tubule and then transferred to the systemic circulation [63]. Disturbances in this process result in glomerular damage, inducing proteinuria and FGF23 upregulation [61]. It has been shown that in patients with T2DM, the use of losartan, a blocker of the binding of angiotensin II (ANGII) to its AT_1_R receptor reduced proteinuria and increased Klotho levels, demonstrating the inverse association between these two factors [64]. Additionally, increased Klotho protein expression is beneficial for regenerative tissue response, besides attenuation of proteinuria, secondary hyperparathyroidism, vascular calcification, and ROS damage, improving health span in the kidney disease context. Furthermore, blocking angiotensin-converting enzyme 1 (ACE-1) with ramipril also decreased proteinuria and concomitantly reduced FGF23 levels [62]. The cross-talk between FG23, Kloto, and ACE-2 may explain the AT_1_R and AT_2_R receptor responses in this study. Therefore, the increase in Klotho levels accompanied by a decrease in FGF23 induced by AT seems to be an important therapeutic target for renal damage attenuation inherent to obesity conditions.

Obese Zucker animals submitted to AT have shown a reduction in asymmetric dimethylarginine (ADMA) and bradykinin (BK) levels, when compared to the CTL-obese group and animals receiving L-NAME (Table 2), which reflect an altered response to blood pressure. Since ADMA is a potent endogenous inhibitor of eNOS and its concentration is inversely correlated with NO^−^ levels [55], a reduction in ADMA can contribute to increase NO^−^ levels in AT obese animals. Gamboa et al. showed that ACE-1 inhibitor (ramipril) increased ADMA levels in patients with kidney disease and appears to have been modulated by bradykinin (BK) during ACE-1 inhibition [65]. Here, we have shown that AT decreased BK and ADMA levels in the kidney, and such adjustments were important to increase NO^−^ and, consequently, renal morphological integrity maintenance. These molecular pathways collectively highlight the importance of NO^−^ on kidney cytoprotection and suggest AT as a prerequisite for the protection of cells from oxidative stress and the support of an elongated life span.

The results of the present study agree with a prior investigation that reported that iNOS inhibition reduced proinflammatory markers and prooxidant agents while improving glycemic homeostasis and kidney integrity in a model of obesity-induced kidney disease [66]. Interestingly, we observed an increase in iNOS mRNA levels in the injured kidneys of obese rats, possibly induced by an increase in proinflammatory cytokines (Table 2). We observed that L-NAME administration can induce a downregulation of eNOS mRNA levels and high expression of iNOS mRNA in the kidney. This condition is associated with increased oxidative stress, promoting a vicious cycle and, consequently, exacerbating tissue damage [27–29, 67, 68].

AT reduced the expression of proteins from the renin-angiotensin system (RAS) in the kidney, representing promising candidates to transduce exercise-induced kidney health benefits (Figure 5). The ANGII increases ADMA levels by stimulating AT_1_R receptors and increasing oxidative stress, which is an important promoter of ADMA synthesis [69]. Angiotensin I (ANGI) is cleaved by ACE-1 forming ANGII, which interacts with AT_1_R receptors and increases of vasodeleterious pathway activity. Additionally, this condition induces ROS formation, inflammation, and insulin resistance [69–71]. On the other hand, glucose metabolism impairments may be restored by exercise via several mechanisms including improvements in insulin secretion and action [72, 73]. These adaptations are essential to maintain enzymatic activity in kidneys, possibly preventing impairments in mitochondrial oxidative capacity.

**Figure 7:**
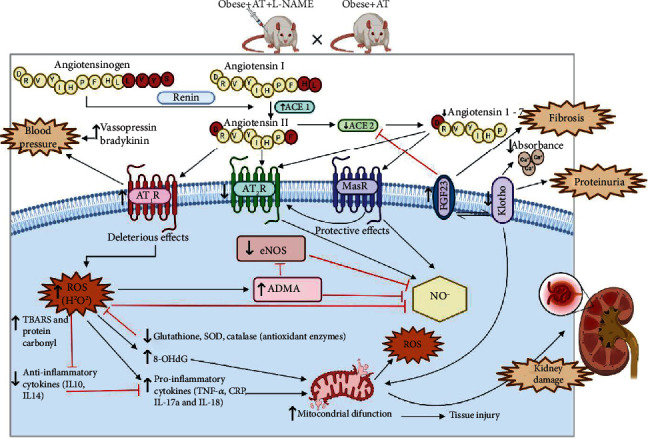
Mechanisms of vasoactive peptide integration. Angiotensinogen is transformed by renin in ANGI, which is cleaved by ACE-1 forming ANGII, interacting on AT_1_R increasing intraglomerular pressure, renal fibrosis, mesangial proliferation, proteinuria, vasopressin release, decreased sodium excretion, and activation of the NOX complex, which induces ROS, inflammation, and insulin resistance, which are common complications of cardiometabolic diseases [58, 71, 81]. However, AT decreases ACE-1 and AT_1_R in the kidney, which was partly responsible for renoprotection. In addition, AT increased ACE-2, which converts ANGII to ANG-(1-7) that interact with MasR and/or ANGII acting on AT_2_R to promote a vasodilatory, antifibrotic, antioxidant, and anti-inflammatory effect, as well as an increase in sodium excretion and maintenance of intrarenal hemodynamics. C-EX: chronic exercise training; ACE-1: angiotensin-converting enzyme 1; ACE-2: angiotensin-converting enzyme 2; AT_1_R: angiotensin II type-1 receptor; AT_2_R: angiotensin II type-2 receptor; MasR: receptor MasR; ROS: reactive oxygen species; ADMA: asymmetric dimethylarginine; NO: nitric oxide.

AT decreased the expression of ACE-1 and AT_1_R, while there was an increase in ACE-2. This is particularly important as ACE-2 converts ANGII to ANG-(1-7), which interacts with MasR. ANGII also acts on AT_2_R receptors that have a critical vasodilator and antifibrotic, anti-inflammatory, and antioxidant effects [74]. Moreover, it has been established that ACE-2 deficiency in mice induces lower physical fitness, suggesting that this enzyme is crucial to mediate improvements in aerobic capacity [75]. Thus, the lower ANGII content and the higher renal ANG-(1-7) promoted by AT partly explain renal injury attenuation, blood pressure adjustments, and aerobic performance.  Figure 7 demonstrates the possible mechanisms involved in the results of the present study.

Furthermore, the decrease in oxidative stress and mitochondrial structure maintenance induced by AT contributed to eNOS regulation (Figure 2 and Table 2) which is an important factor in preventing the progression of kidney injury [67, 76]. We also observed a downregulation in iNOS mRNA levels after AT. This possibly due to a lower proinflammatory cytokine level, such as TNF-*α*, C-reactive protein, IL-17a, and IL-18, which are potent stimulators of iNOS activity [77–80]. Furthermore, there was also an increase in key anti-inflammatory cytokine levels, such as IL-10 and IL-4 after AT, which is also essential to reduce kidney damage. Collectively, these results may have important clinical implications for human studies, as AT is key to control the inflammatory response. However, AT effects upon the kidney seems to be, at least in part dependent on NO^−^ downstream pathways and the mutual crosstalk between oxidative stress and inflammatory responses. More studies, especially in humans, are necessary to improve our understanding of the clinical implications of AT upon the kidney in obesity related disorders, such as T2DM.

## 5. Conclusion

In obese Zucker rats, the renal and metabolic benefits promoted by AT are dependent on NO^−^ bioavailability and its underlying regulatory mechanisms, including the structure and production of mitochondrial ROS, redox balance, inflammation, the Klotho/FGF23 axis, ACE-1/ANGII/AT_1_R, and ACE-2/ANGII/AT_2_R-MasR. Under conditions of low NO^−^ bioavailability, AT can increase the kidney damage by promoting oxidative stress and inflammation, which contributes to the loss of glucose homeostasis already observed in obesity associated T2DM.

## Figures and Tables

**Table 1:** Responses of body weight, blood pressure, metabolic parameters and physical fitness pre- and posttraining.

Variables		CTL-Lean *N* = 10	CTL-Obese *N* = 10	Obese+AT *N* = 10	Obese+AT+L-NAME *N* = 10	Two-way ANOVA		
						*p* group	*p* time	*p* interaction
BW (g)	Pre	384 ± 12	534 ± 9^∗^	533 ± 11	536 ± 13	<0.0001	<0.0001	0.0005
	Post	403 ± 11^§^	569 ± 6^§,∗^	571 ± 11^§^	564 ± 10^§^			
SBP (mmHg)	Pre	117 ± 7	141 ± 8^∗^	143 ± 4	155 ± 6^†,‡^	<0.0001	<0.0001	<0.0001
	Post	121 ± 9	173 ± 5^§,∗^	148 ± 8^†^	152 ± 7^†^			
FBG (mg/dL)	Pre	98 ± 11	141 ± 15^∗^	137 ± 14	158 ± 9^†,‡^	<0.0001	<0.0001	<0.0001
	Post	105 ± 11	211 ± 16^§,∗^	104 ± 11^§,†^	285 ± 45^§,†,‡^			
FPI (ng/mL)	Pre	1.35 ± 0.22	9.58 ± 0.30^∗^	9.49 ± 0.19	9.81 ± 0.12^†,‡^	<0.0001	<0.0001	0.0002
	Post	1.37 ± 0.12	9.67 ± 0.11^∗^	9.13 ± 0.11^§,†^	9.90 ± 0.06^†,‡^			
GTT (AUC)	Pre	900 ± 95	1626 ± 216^∗^	1618 ± 145	1798 ± 67^†,‡^	<0.0001	<0.0001	<0.0001
	Post	912 ± 79	1673 ± 94^∗^	1380 ± 50^§,†^	1964 ± 73^§,†,‡^			
ITT (AUC)	Pre	612 ± 51	759 ± 47^∗^	755 ± 41	935 ± 24^†,‡^	<0.0001	<0.0001	<0.0001
	Post	641 ± 31	837 ± 46^§,∗^	698 ± 34^§,†^	995 ± 40^§,†,‡^			
HOMA-IR	Pre	0.32 ± 0.06	2.70 ± 0.86^∗^	2.98 ± 0.25	3.82 ± 0.21^†,‡^	<0.0001	<0.0001	<0.0001
	Post	0.35 ± 0.05	3.02 ± 0.19^§,∗^	2.40 ± 0.22^§,†^	4.26 ± 0.19^§,†,‡^			
HOMA-*β*	Pre	0.29 ± 0.06	1.91 ± 0.64^∗^	1.60 ± 0.17	1.28 ± 0.07^†,‡^	<0.0001	<0.0001	<0.0001
	Post	0.27 ± 0.04	1.58 ± 0.10^§,∗^	1.78 ± 0.16^§,†^	1.16 ± 0.04^§,†,‡,§^			
*Parameters related to aerobic fitness (m/min)*								
MLSS	Pre	16 ± 2	12 ± 4^∗^	12 ± 3	11 ± 5	<0.0001	0.2491	0.0308
	Post	15 ± 7	11 ± 2^∗^	15 ± 4^§,†^	10 ± 2^‡^			
Vmax	Pre	23 ± 3	14 ± 4^∗^	16 ± 3	14 ± 2^†^	<0.0001	0.3613	<0.0001
	Post	20 ± 6	14 ± 6^∗^	26 ± 5^§,†^	11 ± 3^‡^			

Data are presented as the mean ± SD. CTL-Lean: sedentary lean rats; CTL-Obese: sedentary obese rats; Obese+AT: obese rats who underwent aerobic training without blocking the nitric oxide synthases; Obese+AT+L-NAME: obese rats who underwent aerobic training with blocking the nitric oxide synthases; BW: body weight; SBP: systolic blood pressure; FBG: fasting blood glucose; FPI: fasting plasma insulin; GTT: glucose tolerance test; ITT: insulin tolerance test; AUC: area under the curve; HOMA-IR: homeostasis model assessment for insulin resistance; HOMA-*β*: homeostasis model assessment for *β*-cell function; MLSS: maximal lactate steady state; Vmax: maximum running velocity. Two-way ANOVA, including within and between groups analysis followed by Tukey's post hoc test, was adopted to compare the responses of variables pre- and postexperimental period. § *vs*. Pre; ∗*vs*. CTL-Lean; † *vs*. CTL-Obese; ‡ *vs*. Obese+AT.

**Table 2:** Renal morphology, nitric oxide metabolism, and inflammation.

Variables	CTL-Lean *N* = 10	CTL-Obese *N* = 10	Obese+AT *N* = 10	Obese+AT+L-NAME *N* = 10	*p* value
*Renal morphology and protein excretion*					
Kidney weight (g)	1.67 ± 0.12	2.12 ± 0.18^∗^	2.14 ± 0.25	2.17 ± 0.24	<0.0001
KW/TL (mg/cm)	307 ± 22	389 ± 34^∗^	392 ± 53	398 ± 50	<0.0001
UPE (mg/24 h)	25 ± 8	153 ± 52^∗^	72 ± 31^†^	192 ± 49^‡^	<0.0001
*Molecules related to the metabolism of renal nitric oxide*					
NO^−^ (*μ*mol/mg protein)	14.6 ± 2.2	6.8 ± 1.6^∗^	10.8 ± 1.1^†^	3.7 ± 1.3^†,‡^	<0.0001
ADMA (ng/mg protein)	1.0 ± 0.3	2.1 ± 0.3^∗^	1.4 ± 0.1^†^	2.6 ± 0.2^†,‡^	<0.0001
eNOS (2^-*ΔΔ*CT^)	1.12 ± 0.23	0.40 ± 0.31^∗^	1.35 ± 0.20^†^	0.13 ± 0.05^†,‡^	<0.0001
iNOS (2^-*ΔΔ*CT^)	1.46 ± 0.19	2.17 ± 0.19^∗^	1.00 ± 0.23^†^	2.35 ± 0.09^‡^	<0.0001
*Renal inflammatory profile (pg/mg protein)*					
TNF-*α*	217 ± 21	538 ± 33^∗^	390 ± 36^†^	611 ± 47^†,‡^	<0.0001
CRP	108 ± 12	223 ± 19^∗^	164 ± 24^†^	275 ± 25^†,‡^	<0.0001
IL-17a	18 ± 5	62 ± 7^∗^	33 ± 10^†^	81 ± 130^†,‡^	<0.0001
IL-18	180 ± 56	665 ± 111^∗^	379 ± 139^†^	862 ± 89^†,‡^	<0.0001
IL-10	29 ± 8	8 ± 4^∗^	21 ± 5^†^	3 ± 2^‡^	<0.0001
IL-4	32 ± 8	15 ± 6^∗^	36 ± 10^†^	6 ± 3^†,‡^	<0.0001

Data are presented as the mean ± SD. CTL-Lean: sedentary lean rats; CTL-Obese: sedentary obese rats; Obese+AT: obese rats who underwent aerobic training without blocking of nitric oxide synthases; Obese+AT+L-NAME: obese rats who underwent aerobic training with blocking of nitric oxide synthases; KW: kidney weight; KW/TL: kidney weight corrected for the tibial length; UPE: urinary protein excretion; NO^−^: nitric oxide; ADMA: asymmetric dimethylarginine; eNOS: renal gene expression endothelial nitric oxide synthase; iNOS: renal gene expression inducible nitric oxide synthase; TNF-*α*: tumor necrosis factor-alpha; CRP: c-reactive protein; IL-17a: interleukin-17a; IL-18: interleukin-18; IL-10: interleukin-10; IL-4: interleukin-4. One-way ANOVA followed by Tukey's post hoc test was adopted to verify the difference between the groups. ∗*vs*. CTL-Lean; † *vs*. CTL-Obese; ‡ *vs*. Obese+AT.

## Data Availability

Data are available upon reasonable request to the corresponding author.
